# Longitudinal study of COPD phenotypes using integrated SPECT and qCT imaging

**DOI:** 10.3389/fphys.2025.1555230

**Published:** 2025-04-25

**Authors:** Frank Li, Xuan Zhang, Alejandro P. Comellas, Eric A. Hoffman, Michael M. Graham, Ching-Long Lin

**Affiliations:** ^1^ Roy J. Carver Department of Biomedical Engineering, University of Iowa, Iowa City, IA, United States; ^2^ IIHR-Hydroscience and Engineering, University of Iowa, Iowa City, IA, United States; ^3^ Department of Mechanical Engineering, University of Iowa, Iowa City, IA, United States; ^4^ Department of Internal Medicine, University of Iowa, Iowa City, IA, United States; ^5^ Department of Radiology, University of Iowa, Iowa City, IA, United States

**Keywords:** CT, SPECT, COPD, ventilation, small airway disease

## Abstract

**Introduction:**

The aim of this research is to elucidate chronic obstructive pulmonary disease (COPD) progression by quantifying lung ventilation heterogeneities using single-photon emission computed tomography (SPECT) images and establishing correlations with quantitative computed tomography (qCT) imaging-based metrics. This approach seeks to enhance our understanding of how structural and functional changes influence ventilation heterogeneity in COPD.

**Methods:**

Eight COPD subjects completed a longitudinal study with three visits, spaced about a year apart. CT scans were performed at each visit and qCT-based variables were derived to measure the structural and functional characteristics of the lungs, while the SPECT-based variables were used to quantify lung ventilation heterogeneity. The correlations between key qCT-based variables and SPECT-based variables were examined.

**Results:**

The SPECT-based ventilation heterogeneity (CV_Total_) showed strong correlations with the qCT-based functional small airway disease percentage (fSAD%_Total_) and emphysematous tissue percentage (Emph%_Total_) in the total lung, based on cross-sectional data. Over the 2-year period, changes in SPECT-based hot spots (TC_Max_) exhibited strong negative correlations with changes in fSAD%_Total_, Emph%_Total_, and the average airway diameter in the left upper lobe, as well as a strong positive correlation with alternations in airflow distribution between the upper and lower lobes.

**Discussion:**

In conclusion, this study found strong positive cross-sectional correlations between CV_Total_ and both fSAD% and Emph%, suggesting that these markers primarily reflect static disease severity at a single time point. In contrast, longitudinal correlations between changes in TC_Max_ and other variables over 2 years may capture the dynamic process of hot spot formation, independent of disease severity. These findings suggest that changes in TC_Max_ may serve as a more sensitive biomarker than changes in CV_Total_ for tracking the underlying mechanisms of COPD progression.

## Introduction

Chronic obstructive pulmonary disease (COPD) is a prevalent respiratory disease that imposes a significant burden on healthcare systems worldwide (Halpin et al., 2022). The Global Initiative for Chronic Obstructive Lung Disease (GOLD) report (Global Strategy For Prevention, 2023) has been continuously updated to better characterize the heterogeneities of COPD and improve treatment outcomes (Ferrera et al., 2021; Vogelmeier et al., 2020). In addition, imaging modalities, such as computed tomography (CT) and single-photon emission computed tomography (SPECT), have been utilized to visualize and quantify regional functional and structural changes associated with various pathological processes in lung diseases, such as emphysema (Israel et al., 2019).

Innovative biomarkers based on quantitative CT (qCT) imaging have been developed to investigate the underlying causes of COPD. These biomarkers assess a wide range of risk factors and associated defects, such as airway-branch variation (Smith et al., 2018), dysanapsis (Smith et al., 2020), and pulmonary vascular dysfunction (Alford et al., 2010; Iyer et al., 2016). The introduction of the qCT-based parametric response map has significantly advanced research on functional small airway disease (fSAD) and emphysema (Emph) (Galbán et al., 2012). Both fSAD and Emph are critical phenotypes in COPD, with the former considered a precursor to the latter (Ostridge et al., 2016). It is suggested that targeting small airways with appropriate treatments may potentially control the progression of both airway and parenchymal diseases in COPD (Singh, 2017). Novel multiscale qCT biomarkers have been developed to capture a wide range of phenotypes at various stages of lung disease (Choi et al., 2015). These imaging-based biomarkers enable the identification of unique structural and functional features within COPD subgroups, exhibiting strong associations with distinct clinical characteristics. For instance, Haghighi et al. applied unsupervised clustering techniques to cross-sectional qCT biomarkers and identified four clinically relevant subgroups among former and current smokers, respectively (Haghighi et al., 2018a; Haghighi et al., 2019a). In addition, Zou et al. analyzed longitudinal qCT biomarkers to study COPD progression (Zou et al., 2021a).

SPECT ventilation imaging allows for the assessment of global and regional lung ventilation by measuring the concentration of a radioactive tracer. This tracer acts as a biomarker for ventilation, provided that the aerosol size of the tracer is small enough to reach the alveoli. De Backer et al. demonstrated a strong correlation between CT-based lobar air volume change and lobar SPECT tracer aerosol concentration with aerosol diameter less than 2 µm (De Backer et al., 2010). They further demonstrated that the hot spots observed on SPECT images corresponded to airway narrowing on CT images. Studies have also demonstrated a correlation between heterogeneities observed on SPECT images and impaired lung functions. Xu et al. found that the coefficient of variation (CV) observed in SPECT images, a measure of ventilation heterogeneity, not only differentiated patients with emphysema from non-emphysematous smokers and non-smokers, but also correlated with pulmonary functions (Xu et al., 2001). Thus, combining SPECT and CT imaging allows for a comprehensive evaluation of both functional and structural relationships within different regions of the lungs (Israel et al., 2019).

In this study, we aimed to evaluate COPD progression by quantifying structural and functional changes in CT and SPECT images of COPD subjects acquired at three visits, approximately 1 year apart. Our objective was to establish connections between qCT-based variables and SPECT-based biomarkers to better understand how structural and functional alterations interact both cross-sectionally and longitudinally during COPD progression, offering new insights into ventilation heterogeneities. We hypothesized that qCT-based structural and functional alterations correlate with SPECT-measured functional ventilation characteristics in COPD. While this correlation was anticipated, it had never been established or quantified *in vivo.*

## Materials and methods

Fourteen subjects were initially recruited for a longitudinal study with three visits at baseline (V0), 12.84 ± 1.68 months (V1), and 26.20 ± 3.06 months (V2). The inclusion criteria were as follows: current or former smokers with at least a 10 pack-year smoking history, who have been classified as a COPD GOLD 0-4. Five subjects dropped out due to other health issues or personal reasons, and one subject was excluded due to an abnormal airway structure with an accessary bronchus connected to the right main bronchus. Thus, eight subjects who completed all three visits were analyzed in this study. Pulmonary function tests (PFTs, see Supplementary Material/Content 1; Supplementary Figure S1 for more details), CT scans, and SPECT scans were acquired at each visit. This study was approved by the institutional review board of the University of Iowa and the informed consent was obtained from all the patients before the study.

### CT images and qCT variables

Three static three-dimensional (3D) CT scans were acquired using the Siemens SOMATOM Force Scanner at the lung volumes of total lung capacity (TLC), functional residual capacity (FRC) and residual volume (RV). The lung volume control system used was the same as that employed in the previous study (Fuld et al., 2012). The TLC scan protocol used dose modulation with 36 reference mAs, 120 kV, pitch of 1.0, and rotation time of 0.25 s. The FRC and RV scans used dose modulation with 15 reference mAs, 120 kV, pitch of 1.0, and rotation time of 0.25 s. The CT images have a spatial resolution of 0.5 mm in the z-axis, and 0.5–0.7 mm in the x and y-axes. The subjects were positioned on the CT table in the supine position and were instructed to breathe normally through the mouthpiece for a few breathing cycles. While the CT scans were performed, they were instructed to take in a deepest breath and hold it at the desired lung volume of TLC, FRC, or RV. The subject breathed normally for several cycles before the next CT scan was performed. Repeatability tests assessing qCT measures have demonstrated excellent reliability and reproducibility (Motahari et al., 2023).

**FIGURE 1:**
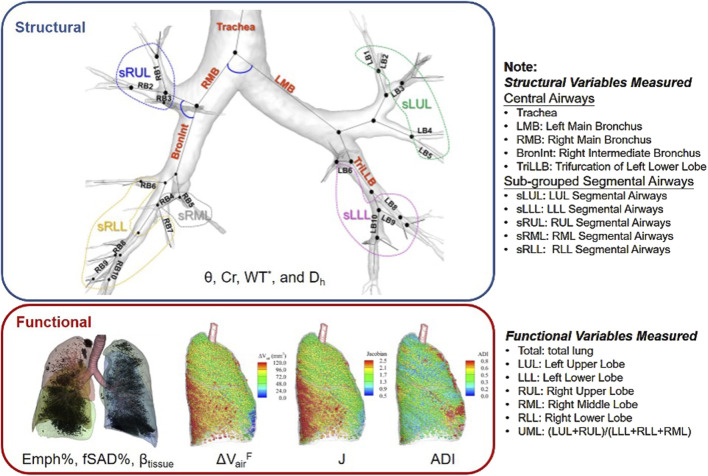
The multiscale structural and functional qCT variables.

The CT images were processed using VIDA Vision (VIDA Diagnostics Inc., Coralville, Iowa) to segment the lung, lobes, and airways. A mass preserving image registration technique was performed to match FRC (or RV) with TLC images (Haghighi et al., 2018b). TLC and RV images were registered to derive functional variables, while TLC and FRC images were registered to match SPECT images. A total of 69 structural and functional multiscale qCT variables presented in previous studies were derived (Haghighi et al., 2018a; Haghighi et al., 2019a; Zou et al., 2021a) as shown in Figure 1. Please refer to Supplementary Material/Content 2 for a detailed explanation of these variables, and to Content 3 for a list of abbreviations of qCT variables. The structural variables describe regional alterations in lung structures, while the functional variables capture changes in regional lung function. The qCT variables most relevant to this study include normalized airway wall thickness (WT^*^), normalized airway hydraulic diameter (D_h_^*^), fractional air volume change ( ${∆V}_{air}^{F}$ ), determinant of Jacobian matrix (J), anisotropic deformation index (ADI), fraction-based small airways disease (fSAD%), fraction-based emphysema (Emph%), and tissue fraction at TLC (β_tissue_). Specifically, the wall thickness and hydraulic diameter were normalized, as indicated by an asterisk, using predicted values from healthy subjects to account for inter-subject variability related to sex, age, and height (Choi et al., 2015). ${∆V}_{air}^{F}$ represents the ratio of the air-volume change in the lobes to the total air-volume change in the whole lung, while ${∆V}_{air,UML}^{F}$ represents the ratio of the air-volume change in the upper lobes to the air-volume change in the combined middle and lower lobes.

The region where the qCT variable was measured is indicated as a subscript of the variable, formatted as {Variable}_{Region}_. The structural variables were measured from the lung shape, central airways, and segmental airways. The lung shape was measured at TLC as the ratio of apical-basal distance and ventral-dorsal distance. The central airways include trachea, left main bronchus (LMB), right main bronchus (RMB), right intermediate bronchus (BronInt), and trifurcation of left lower lobe (TriLLB). The segmental airways were grouped by lobes, such as sub-grouped segmental airways of left upper lobe (sLUL), sub-grouped segmental airways of left lower lobe (sLLL), sub-grouped segmental airways of right upper lobe (sRUL), sub-grouped segmental airways of right middle lobe (sRML), and sub-grouped segmental airways of right lower lobe (sRLL). The functional variables were measured at lobar level, including left upper lobe (LUL), left lower lobe (LLL), right upper lobe (RUL), right middle lobe (RML), and right lower lobe (RLL), as well as total lung level (Total).

### SPECT images and variables

The subject was positioned supine on the SPECT scanner to replicate the positioning used in the above CT scans. The subject inhaled ^99m^Tc sulfur colloid, a radioactive tracer aerosol generated by a specialized nebulizer, through a mouthpiece with the nose occluded. A 3D SPECT scan of the whole lung was then performed. The particle size of the sulfur colloid is below 1 µm (Krogsgaard, 1976), allowing the tracer aerosol to penetrate deep into the lung (Sirr et al., 1985). Since ^99m^Tc sulfur colloid is non-absorbable, it is not rapidly cleared from the lungs. The 3D SPECT scan took approximately 20 min to complete and was acquired during continuous tidal breathing. The SPECT images have a spatial resolution of 3.895 mm along the x, y, and z-axes. The estimated radiation dose retained by the subject was 0.17 mSv, well below the U.S. Nuclear Regulatory Commission standard of 50 mSv/year. Initially, the SPECT imaging protocol involved using ^99m^Tc-HMPAO labeled neutrophils to assess inflammation distribution. This method was used for the first two subjects at V0 but yielded minimal signal capture, as the ^99m^Tc-HMPAO-labeled neutrophils moved too quickly through the pulmonary vessels. Consequently, we modified the protocol, which is why the first two subjects did not have SPECT ventilation scans at V0.

SPECT imaging is a dynamic process that produces time-averaged images during tidal breathing, while CT images are static scans acquired at TLC, FRC, and RV. It is preferable to match the SPECT images with the FRC CT images, as FRC represents a lung volume closer to tidal breathing, compared to RV or TLC. The lung deformation from FRC to the peak of tidal volume was assumed to be relatively small and quasi-linear. Thus, the SPECT images were first aligned with the FRC CT images using affine transforms, with mutual information as the cost function. Displacement fields for transforming the FRC CT images to TLC were then obtained through image registration. These displacement fields were subsequently applied to the SPECT images, deforming them to match the TLC CT images, which provide detailed anatomical information of the lungs.

After aligning the SPECT images with the TLC CT images, tracer concentrations (TC) within the lung and each lobe were quantified based on the domain of TLC CT images. The ratio of TC in a lobe over the total lung (TC%), the TC coefficient of variation for the total lung (CV_Total_), and the standardized maximum TC in the total lung (TC_Max_) were used to quantify the distribution, heterogeneity, and hot spots of TC. Specifically, TC% measured lobar ventilation distribution, while CV_Total_, the ratio of the standard deviation to the average of TC (Xu et al., 2001), measured ventilation heterogeneity. TC_Max_ quantified the magnitude of local hot spots as an indicator of airway narrowing. The normalized CV_Total_ and TC_Max_ accounted for variations in the amount of TC inhaled. Moreover, the correlation between TC% and ${∆V}_{air}^{F}$ was used to assess the efficacy of the image registration of SPECT and CT images. Pearson’s correlation coefficients (r) between CV_Total_ and PFT values were calculated and validated against previous studies (Xu et al., 2001; Nagao and Murase, 2002). Correlation strengths were categorized as follows: |r| ≤ 0.3 as negligible, 0.3 < |r| ≤ 0.5 as weak, 0.5 < |r| ≤ 0.7 as moderate, 0.7 < |r| ≤ 0.9 as strong, and |r| > 0.9 as very strong.

### Analysis of SPECT and qCT variables

The associations between SPECT and qCT variables were evaluated to enhance the interpretation of the SPECT variables. Exploratory factor analysis (EFA) was applied to reduce the extensive number of qCT variables derived from the 799 current and former smokers analyzed previously (Haghighi et al., 2018a; Haghighi et al., 2019a) to a smaller set of factors that preserved the variability of qCT-captured features. EFA identifies patterns or structures in a large set of variables by reducing them to a smaller number of factors, making complex data easier to interpret. The number of factors was determined using the parallel analysis (Horn, 1965). Factors were extracted using principal component analysis with Varimax orthogonal rotation (Kaiser, 1958) to ensure their independence.

We then identified key qCT variables that significantly contributed to each factor, using them as surrogates to interpret disease phenotypes. qCT variables were regarded as key if their loading values on their corresponding factors exceeded 0.6. We then examined the correlations between key qCT variables and SPECT variables, using cross-sectional data from V0, V1, and V2 as well as the longitudinal data showing changes between any two visits of V1 and V0 (V1-V0, 1 year apart), V2 and V1 (V2-V1, 1 year apart), and V2 and V0 (V2-V0, 2 years apart).

With small sample sizes, we prioritized large effect sizes (Pearson’s coefficients >0.7) in this exploratory study (Akoglu, 2018; Cohen, 1992). This approach acknowledges two challenges of small samples: insufficient statistical power to detect real effects (increasing Type II errors) and vulnerability to spurious significance from random fluctuations. Importantly, statistical power can be deemed adequate with a smaller sample when the effect size is large (Shreffler and Huecker, 2023). By focusing on effect size, we quantified relationship magnitudes independent of sample size, ensuring the observed effects were substantial and meaningful despite limited data.

## Result

The clinical data and PFT results for the eight COPD subjects are presented in Table 1 (see Supplementary Table S1 for more data). These subjects (age: 63.1 ± 11.5 years; range: 51–84 years) had an average Tiffeneau ratio [FEV_1_/FVC (%)] of 52.9% ± 16.6% and an average predicted forced expiratory volume ratio (FEV_1_% predicted) of 67.9% ± 16.3% at V0. The mean changes between V1 and V0 were 0.0% ± 1.9% for FEV_1_/FVC (%) and −2.5% ± 5.4% for FEV_1_ (%) predicted. Additionally, the mean changes between V2 and V1 were 0.8% ± 1.9% for FEV_1_/FVC (%) and 3.5% ± 4.6% for FEV_1_% predicted. Thus, the lung function of all subjects remained approximately stable over the 2-year period (Pellegrino et al., 2005).

**TABLE 1:** Clinical data, PFT at V0, and changes in PFT between visits. Subjects 1–5 were former smokers, while Subjects 6–8 were current smokers.

Subject	Sex	Age (yrs.) at V0	BMI	GOLD stage	V0		V1 - V0		V2 - V0	
					FEV1% predicted	FEV1/FVC (%)	ΔFEV1% predicted	ΔFEV1/FVC (%)	ΔFEV1% predicted	ΔFEV1/FVC (%)
1	M	80	32	2	73	53	1	3	3	0
2	M	84	23	2	50	28	−11	−2	1	1
3	M	55	26	2	63	51	−2	0	−1	0
4	M	59	21	2	73	56	−5	2	5	4
5	M	59	32	3	36	28	−5	−3	−3	0
6	M	51	29	1	83	59	−5	−1	−2	0
7	F	54	27	0	77	71	−2	0	−4	0
8	F	63	36	0	88	77	9	1	9	1
Mean ± SD		63.1 ± 11.5	28.3 ± 4.7		67.9 ± 16.3	52.9 ± 16.6	−2.5 ± 5.4	0.0 ± 1.9	1.0 ± 4.2	0.8 ± 1.3

At V0, Subjects 2 and 5 had moderate-to-severe (GOLD 2, bordering on GOLD 3) and severe (GOLD 3) airflow obstruction, respectively, according to their PFT results. Subjects 1, 3, and 4 had moderate (GOLD 2) airflow obstruction. Subject 6 had mild airflow obstruction (GOLD 1), while Subjects 7 and 8 were at risk of COPD (GOLD 0). Additionally, Subjects 1 through 5 were former smokers, while Subjects 6 through 8 were current smokers.

### SPECT features

**FIGURE 2:**
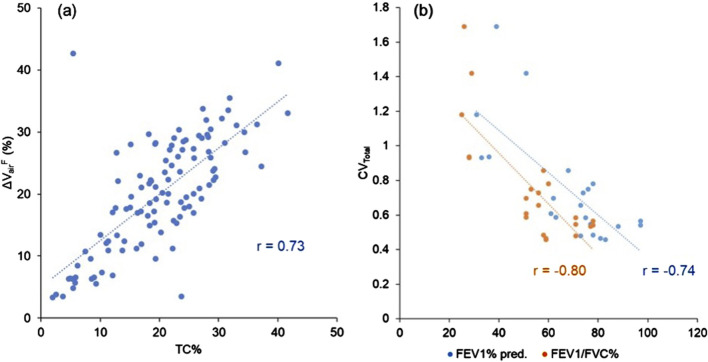
**(a)** A scatter plot showing the strong correlation between TC% and ΔV_air_^F^ of the lobes (r = 0.73). **(b)** A scatter plot showing the strong correlations between CV_Total_ and PFT results (FEV_1_% predicted: r = −0.74, FEV_1_/FVC (%): r = −0.80). Data points are derived from all three visits.

TC% and ${∆V}_{air}^{F}$ of each lobe at each visit are listed in Supplementary Table S2. The correlation coefficient between TC% and ${∆V}_{air}^{F}$ was 0.73, indicating a strong positive relationship between the two variables (Figure 2a). In addition, the correlations between CV_Total_ and PFT values revealed a strong negative correlation with FEV_1_% predicted (r = −0.74) and an even stronger negative correlation with FEV_1_/FVC (%) (r = −0.80) (Figure 2b).

**FIGURE 3:**
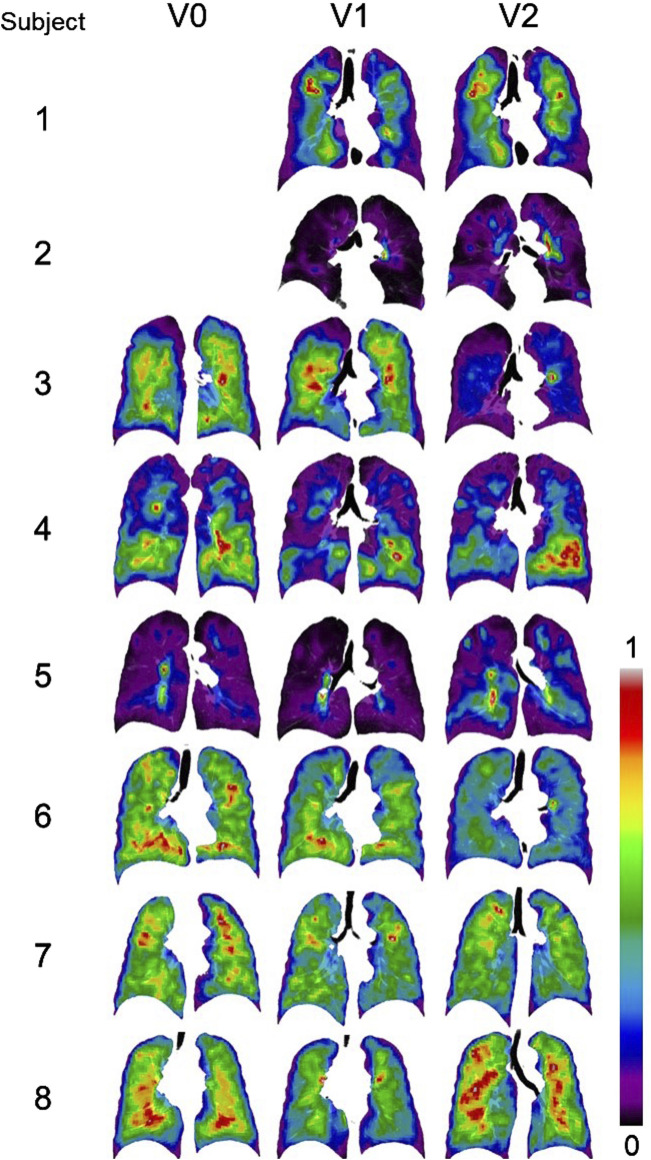
Normalized SPECT images transformed to the TLC domain at three visits. Subjects 1 and 2 did not have ventilation scans at V0. Intensities were normalized using the minimum and maximum values, resulting in contour values ranging from 0 to 1. The localized red spots in Subjects 2 and 5 indicate high-intensity hot spots where inhaled aerosols tend to deposit.

The SPECT images for all the subjects are displayed in Figure 3. CV_Total_ and TC_Max_ at each visit, together with their changes between visits, are summarized in Table 2. Across all visits, these subjects were categorized into three distinct subgroups: (Global Strategy for Prevention, 2023; Israel et al., 2019), (Smith et al., 2018; Smith et al., 2020; Alford et al., 2010) and (Halpin et al., 2022; Ferrera et al., 2021; Vogelmeier et al., 2020). Subjects 2 and 5 were characterized by higher CV_Total_ and extremely elevated TC_Max_. In contrast, Subjects 6, 7, and 8 exhibited more homogeneous TC distributions, while Subjects 1, 3, and 4 showed ventilation distribution patterns that fell between the other two subgroups. Notable hot spots, revealed by concentrated red areas, were evident in the SPECT images of Subjects 2 and 5 throughout all visits. A prominent hot spot was also observed on the left upper lobe in the SPECT image of Subject 3 at V2. The changes of CV_Total_ and TC_Max_ between visits were minimal for all subjects, around 10%.

**TABLE 2:** CV_Total_ and TC_Max_ at each visit and their changes between visits.

Subject	V0	V1	V2	V1-V0	V2-V1	V2-V0
CV_Total_						
1		0.73	0.75		0.02	
2		1.69	1.42		−0.27	
3	0.59	0.61	0.7	0.02	0.09	0.11
4	0.66	0.86	0.78	0.2	−0.08	0.13
5	0.94	1.18	0.93	0.24	−0.25	0
6	0.46	0.48	0.47	0.03	−0.02	0.01
7	0.55	0.58	0.48	0.04	−0.1	−0.07
8	0.53	0.57	0.54	0.03	−0.02	0.01
Mean ± SD	0.62 ± 0.15	0.84 ± 0.38	0.76 ± 0.29	0.09 ± 0.09	−0.08 ± 0.12	0.03 ± 0.07
TC_Max_						
1		7.27	5.36		−1.91	
2		37.69	20.83		−16.86	
3	7.32	5.36	16.33	−1.97	10.97	9.01
4	5.03	14.22	7.82	9.19	−6.4	2.79
5	24.1	18.66	19.59	−5.44	0.93	−4.51
6	3.51	4.19	9.23	0.68	5.05	5.72
7	4.14	5	4.2	0.86	−0.81	0.06
8	3.82	4.21	3.55	0.39	−0.65	−0.26
Mean ± SD	7.99 ± 7.31	12.07 ± 10.88	10.87 ± 6.57	0.62 ± 4.41	−1.21 ± 7.62	2.14 ± 4.37

### Key qCT-based factors and variables

Seven factors, extracted from the qCT variables of former and current smokers (Choi et al., 2015; Haghighi et al., 2018a), accounted for 78.4% of the variance in the original features. Among all the qCT variables, ADI_Total_, J_Total_, β_tissue_,_Total_, fSAD%_Total_, D_h_^*^_sLUL_, Emph%_Total_, ∆ V^F^_air,UML_, WT^*^_sLLL_, and D_h_^*^_LMB_ were the key qCT variables that contributed significantly to the factors and were selected for analysis in this study. The key qCT variables with moderate-to-high (factor loading = 0.6–0.7) and high contributions to the factors (factor loading >0.7) are listed in Supplementary Table S2.

The above key qCT variables at each visit and their changes between visits are presented in Table 3; Supplementary Table S3. Across all visits, Subjects 2 and 5 exhibited higher fSAD%_Total_ and Emph%_Total_ compared to the other subjects. Subjects 1, 3, and 4 showed moderate fSAD%_Total_, while Subjects 6 through 8 had low fSAD%_Total_. Within the subgroups (Halpin et al., 2022; Ferrera et al., 2021; Vogelmeier et al., 2020) and (Smith et al., 2018; Smith et al., 2020; Alford et al., 2010), Emph%_Total_ was mild for all subjects except Subject 3.

**TABLE 3:** The selected qCT variables at each visit and their changes between visits.

Subject	V0	V1	V2	V1-V0	V2-V1	V2-V0
fSAD%_Total_						
1	12.49	14.3	12.52	1.81	−1.78	0.03
2	34.01	32.71	31.15	−1.3	−1.56	−2.86
3	13.55	13.13	12.26	−0.42	−0.87	−1.29
4	11.29	11.13	8.46	−0.16	−2.67	−2.84
5	22.83	21.3	24.86	−1.53	3.55	2.03
6	1.36	0.93	0.64	−0.43	−0.3	−0.73
7	0.65	0.8	0.35	0.16	−0.45	−0.3
8	0.53	0.96	1.37	0.43	0.41	0.84
Mean ± SD	12.09 ± 11.06	11.91 ± 10.56	11.45 ± 10.73	−0.18 ± 0.98	−0.46 ± 1.76	−0.64 ± 1.58
*D*_h,sLUL_*						
1	0.21	0.21	0.21	0	0	0
2	0.23	0.18	0.23	−0.05	0.05	0
3	0.23	0.14	0.22	−0.09	0.09	−0.01
4	0.21	0.2	0.21	−0.01	0.01	0
5	0.25	0.21	0.31	−0.03	0.1	0.06
6	0.28	0.27	0.25	−0.01	−0.01	−0.02
7	0.26	0.3	0.24	0.04	−0.06	−0.02
8	0.22	0.24	0.25	0.02	0.01	0.02
Mean ± SD	0.24 ± 0.02	0.22 ± 0.05	0.24 ± 0.03	−0.02 ± 0.04	0.02 ± 0.05	0 ± 0.03
Emph%_Total_						
1	3.15	7.57	8.47	4.42	0.9	5.32
2	27.12	29.64	29.37	2.52	−0.27	2.25
3	18.14	18.65	15.93	0.51	−2.72	−2.21
4	3.43	3.31	3.88	−0.12	0.58	0.46
5	39.36	40.42	41.1	1.06	0.68	1.74
6	3.39	4.25	4.32	0.86	0.06	0.92
7	0.9	0.65	0.75	−0.25	0.1	−0.15
8	4.97	6.51	7.07	1.53	0.56	2.1
Mean ± SD	12.56 ± 13.28	13.87 ± 13.46	13.86 ± 13.34	1.32 ± 1.44	−0.01 ± 1.09	1.3 ± 2.04
${∆V}_{air,UML}^{F}$						
1	0.54	0.46	0.54	−0.08	0.08	0
2	1.43	1.68	1.91	0.25	0.24	0.48
3	1.44	1.59	2.61	0.14	1.02	1.17
4	0.61	0.62	0.7	0.02	0.08	0.1
5	1.22	1.3	1.2	0.07	−0.09	−0.02
6	0.92	0.85	1	−0.08	0.15	0.08
7	0.6	0.59	0.7	−0.01	0.11	0.1
8	0.55	0.54	0.56	−0.01	0.02	0.01
Mean ± SD	0.91 ± 0.37	0.95 ± 0.46	1.15 ± 0.69	0.04 ± 0.1	0.2 ± 0.32	0.24 ± 0.38

### Association between SPECT and qCT variables

**FIGURE 4:**
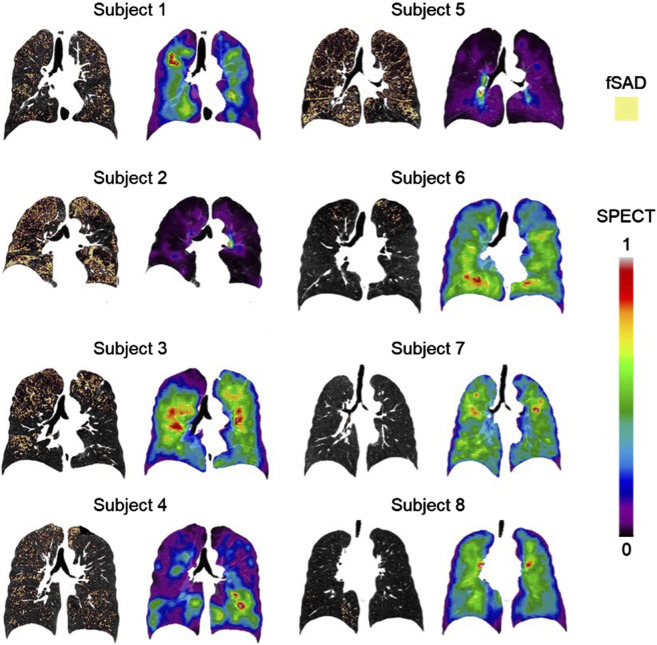
The fSAD-voxel maps (left column) and normalized TC SPECT images (right column) for each subject at V1, where fSAD stands for functional small airway disease and TC represents tracer concentration. These images were plotted in the coronal planes of the CT images. The relationship between qCT-based fSAD maps and SPECT-based ventilation patterns reveals distinct groupings among subjects: Subjects 2 and 5 exhibit high fSAD%_Total_, Subjects 1, 3, and 4 display moderate fSAD%_Total_, and Subjects 6, 7, and 8 demonstrate low fSAD%_Total_. SPECT intensities were normalized using the minimum and maximum values, resulting in contour values ranging from 0 to 1.

Association tests between SPECT and qCT variables were conducted using both cross-sectional and longitudinal data. The cross-sectional tests captured inter-subject variation, while the longitudinal tests tracked intra-subject progression. In the cross-sectional analysis, the SPECT variables CV_Total_ and TC_Max_ showed the strongest correlations with the qCT variables fSAD%_Total_ and Emph%_Total_. Specifically, CV_Total_ correlated 0.90 with fSAD%_Total_ and 0.71 with Emph%_Total_. Similarly, TC_Max_ correlated 0.86 with fSAD%_Total_ and 0.77 with Emph%_Total_. This association is visually illustrated in Figure 4, which compares the fSAD-pixel maps on CT coronal slices at V1 with TC distributions in the SPECT images. The relationship between qCT-based fSAD maps and SPECT-based ventilation patterns is particularly evident in the grouping of Subjects (Global Strategy for Prevention, 2023; Israel et al., 2019), (Halpin et al., 2022; Ferrera et al., 2021; Vogelmeier et al., 2020), and (Smith et al., 2018; Smith et al., 2020; Alford et al., 2010), which correspond to high, moderate, and low fSAD%_Total_, respectively. Importantly, participants who had more compromised lung function (specifically participants 2, 3, and 5) showed ${∆V}_{air,UML}^{F}$ values exceeding 1 throughout all visits, as indicated in Table 3.

**FIGURE 5:**
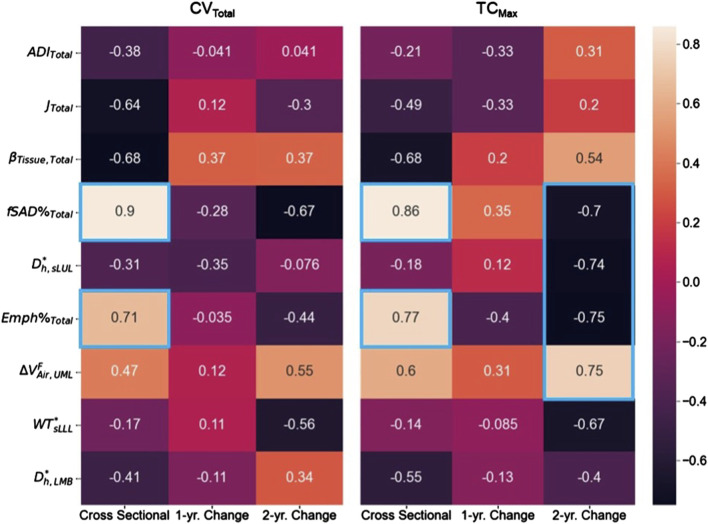
The correlation maps between the SPECT variables and the qCT variables for both cross-sectional and longitudinal data. Correlation magnitudes exceeding 0.7 were highlighted using blue boxes.

In the longitudinal analysis, the correlations between the 1-year changes in SPECT variables and the corresponding changes in qCT variables were weak. However, over 2 years, TC_Max_ exhibited strong correlations with several qCT variables, including fSAD%_Total_ (r = −0.70), D_h,sLUL_^*^ (r = −0.74), Emph%_Total_ (r = −0.75), and ${∆V}_{air,UML}^{F}$ (r = 0.75) (Figure 5). Additionally, the change (V2-V0) in D_h,sLUL_^*^ showed a moderate correlation with the change in fSAD%_Total_ (r = 0.61), while the change (V2-V0) in ${∆V}_{air,UML}^{F}$ was moderately correlated with the change in Emph%_Total_ (r = −0.63) (Supplementary Figure S2).

**FIGURE 6:**
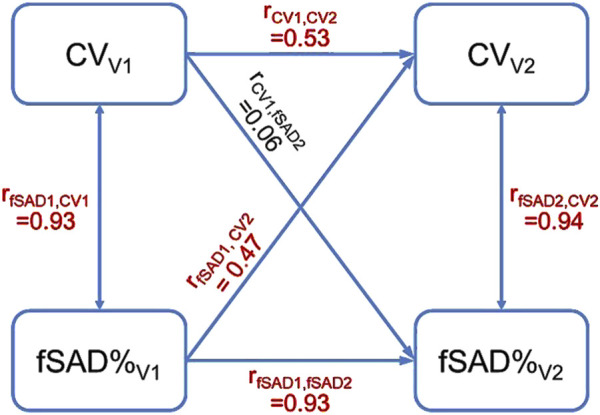
The cross-lagged panel analysis estimated a total of six correlations. The correlations colored in red were significantly greater than zero. The result that r_fSAD1,CV2_ was greater than zero suggested that fSAD cause the heterogeneity of lung ventilation.

A *post hoc* study was conducted to establish the causal relationship between fSAD and heterogeneity of lung ventilation. Cross-lagged panel analysis, which is commonly used to infer the direction and strength of a relationship between two variables measured repeatedly at different time points, was employed (Kenny, 1975). The imaging variables of fSAD%_Total_ and CV_Total_ at V1 and V2 were selected for analysis since they were strongly correlated at all visits. The type one error rate (α) was set at 0.05. As demonstrated in Figure 6, two synchronous correlations (r_fSAD1,CV1_ and r_fSAD2,CV2_) and two stability correlations (r_CV1,CV2_ and r_fSAD1,fSAD2_) were significantly greater than zero, indicating that the assumptions of synchronicity and stationarity were not violated. For the cross-lagged correlations (r_fSAD1,CV2_ and r_CV1,fSAD2_), r_fSAD1,CV2_ was significantly greater than zero while r_CV1,fSAD2_ was not, suggesting that fSAD is the cause of heterogeneity of lung ventilation. Note that the cross-lagged correlations were partial correlations, with the contributions of the stability correlations partialled out. In COPD, the narrowing of small airways leads to higher airflow resistance and greater particle deposition in the lungs (Marsh et al., 2006; Kim and Kang, 1997; Zhang et al., 2022a). As a result, subjects with higher fSAD%_Total_ were observed to have localized areas of elevated TC activity.

## Discussion

This longitudinal study successfully enrolled eight COPD subjects for three visits to examine cross-sectional and longitudinal relationships between qCT and SPECT biomarkers in COPD. Cross-sectional relationships reflect associations between variables at a single time point, whereas longitudinal relationships describe changes in these variables over time. Our analysis revealed strong positive cross-sectional correlations between SPECT-measured ventilation heterogeneity (CV_Total_) and qCT-derived functional small airway disease (fSAD%_Total_) and emphysema (Emph%_Total_), suggesting that both small airway abnormalities and parenchymal destruction contribute to ventilation abnormalities in COPD, reflecting disease severity. Longitudinally, changes in TC_Max_ exhibited strong negative correlations with changes in fSAD%_Total_, Emph%_Total_, and average airway diameter, as well as a strong positive correlation with changes in lobar airflow distribution. These findings suggest that longitudinal changes in TC_Max_ may serve as a more sensitive biomarker for capturing the dynamic process of hot spot formation, independent of disease severity.

To our knowledge, this study is the first longitudinal CT/SPECT analysis to provide insights into COPD progression. The novelty of this study lies in its comprehensive qCT-based phenotyping at both local (segmental) and global (whole-lung) scales, the integration of dual imaging modalities, and its longitudinal design.

### Association between imaging variables and PFTs

The strong correlation observed between TC% and ${∆V}_{air}^{F}$ of the lobes (Figure 2: r = 0.73) indicated that qCT-based ${∆V}_{air}^{F}$ can serve as a viable alternative for measuring lobar ventilation. This correlation also validates the effectiveness of the registration process utilized to align SPECT and CT images. Furthermore, significant negative correlations were found between TC heterogeneity (CV_Total_) and PFT results (Figure 2: r = −0.74 with FEV_1_% predicted; r = −0.80 with FEV_1_/FVC (%)), agreeing with previous studies (r = −0.84 to −0.89 with FEV_1_% predicted) (Xu et al., 2001; Doganay et al., 2019).

### Association between SPECT and qCT variables

The SPECT variables CV_Total_ and TC_Max_ showed strong correlations with the qCT variables fSAD%_Total_ (CV_Total_: r = 0.90; TC_Max_: r = 0.86) and Emph%_Total_ (CV_Total_: r = 0.71; TC_Max_: r = 0.77) in the cross-sectional data. This indicates that both small airway disease and emphysema are associated with lung ventilation heterogeneity, with small airway disease showing a stronger correlation.

**FIGURE 7:**
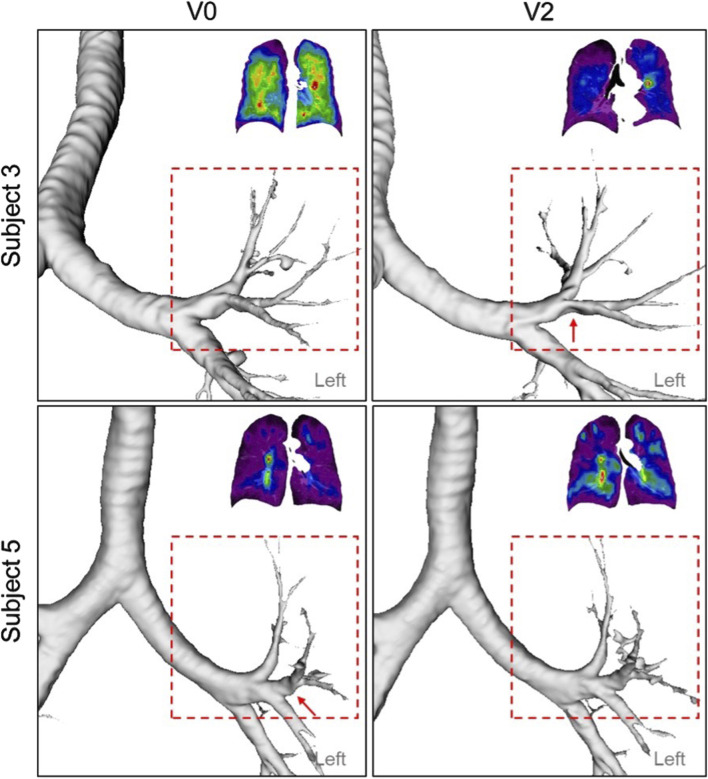
A constricted segmental airway in the LUL (red arrow) of Subject 3 at V2, which may contribute to the TC hot spot observed in the same region. In addition, a constricted segmental airway in the LUL (red arrow) of Subject 5, present at V0, had resolved by V2, potentially increasing ventilation homogeneity.

Analysis of the longitudinal data collected over 2 years revealed strong correlations between TC_Max_ and D_h,sLUL_^*^ (r = −0.74) as well as ΔV_air_^F^_UML_ (r = 0.75) (Figure 5). These findings suggest that reduced diameters of segmental airways and imbalances in ventilation between the upper and lower lobes may contribute to the development of TC hot spots. In human anatomy, the lower lobes of the lungs are generally larger than the upper lobes in terms of volume (Yamada et al., 2020). Notably, subjects with more impaired lung function (subjects 2, 3, and 5) consistently demonstrated ${∆V}_{air,UML}^{F}$ values greater than 1 across all visits (Table 3), suggesting compensatory mechanisms where ventilation shifts preferentially to the upper lobes rather than the lower lobes. Visual inspection of the segmental airways in the LUL of Subjects 3 and 5 showed constricted branches at V2 and V0, respectively (Figure 7). This observation highlights the negative correlations between changes in D_h,sLUL_^*^ and changes in SPECT ventilation images, supporting a previous study identifying D_h,sLUL_^*^ as a significant qCT variable in characterizing COPD subjects (Haghighi et al., 2019a).

Furthermore, changes in TC_Max_ over 2 years were negatively correlated with changes in fSAD% (r = −0.70) and Emph% (r = −0.75) (Figure 5). This suggests that variations in D_h,sLUL_^*^ and ${∆V}_{air,UML}^{F}$ may moderate the relationship between changes in TC_Max_ and changes in fSAD%_Total_ and Emph%_Total_, particularly when disease progression, in terms of COPD stages, remains relatively stable. Namely, Emph%_Total_ and fSAD%_Total_ progression may experience airway collapse/alveolar destruction, which contribute less to overall ventilation and redistribute airflow to less affected areas. These findings indicate that the formation of hot spots in COPD patients could be influenced by alterations in segmental airways in the left upper lobe (Figure 7), as well as changes in airflow distribution between the upper and lower lobes, in addition to factors such as fSAD and emphysema.

Interestingly, the correlations between TC_Max_ and both fSAD% and Emph% in the longitudinal data differed from those in the cross-sectional data. Cross-sectional correlations reflect static disease phenotypes and severity at a single time point, whereas longitudinal correlations capture changes in variables over 2 years, independent of severity. These findings align with Zou et al. (2021a)’s distinction between cross-sectional and longitudinal analyses, where “cross-sectional clustering was based on static disease stage (severity)” while “longitudinal clustering aims to identify COPD progression clusters, which are more dependent upon disease progression than severity.” In our data, cross-sectional correlations capture disease severity, with increased small airway disease and emphysema leading to greater ventilation heterogeneity. However, longitudinally, areas with high TC_Max_ may experience reduced progression of fSAD and emphysema, possibly due to compensatory mechanisms or a plateau in disease progression. These findings suggest that studies investigating COPD progression should consider not only severity markers but also progression patterns identified through longitudinal imaging.

The U.S. National Institutes of Health (NIH) has sponsored multi-center trials, such as the Genetic Epidemiology of COPD study (COPDGene) (Regan et al., 2010; Regan et al., 2019) and the SubPopulations and InteRmediate Outcome Measures in COPD Study (SPIROMICS) (Couper et al., 2014; Woodruff et al., 2015), to collect comprehensive clinical, biologic, genetic, and CT data across large populations. These efforts aim to uncover novel disease pathways, identify surrogate markers of severity, define endotypes, predict health trajectories, and inform clinical trial and treatment strategies. Additionally, these large datasets present both opportunities and challenges for advancing analytical, computational, and machine learning methods in complex biological systems (Peng et al., 2021; Alber et al., 2019; Lin et al., 2021). For example, based on COPDGene data, several novel COPD subgroups and pathways have been identified (Young et al., 2019a; Young et al., 2019b), including an airway-predominant disease subgroup progressing from GOLD 0 to preserved ratio-impaired spirometry (PRISm) status, and an emphysema-predominant disease subgroup progressing from GOLD 0 to GOLD 1 status.

Furthermore, based on SPIROMICS data, qCT-based clusters have been identified in both current and former smokers (Haghighi et al., 2018c; Haghighi et al., 2019b), with additional longitudinal qCT-based clusters identified in former smokers (Zou et al., 2021b). Deep learning approaches have also been developed to identify lung tissue pattern clusters, their latent traits, and associations with drug use in COPD patients (Li et al., 2021). In addition, a CT-based, subject-specific computational fluid and particle dynamics (CFPD) model for the whole lung has also been developed and applied to post-COVID-19 subjects to explore airway resistance and particle deposition across different subgroups (Zhang et al., 2022b; Zhang et al., 2024).

The ultimate goal of this study is to bridge the gaps between big data (including clinical and imaging data), artificial intelligence (machine learning and deep learning), and advanced computational models. The specific objective was to explore the connections between qCT-based variables and SPECT-measured ventilation features, both cross-sectionally and longitudinally. In future studies, the findings summarized below can be used to assess the sensitivity of the CFPD model in predicting SPECT-measured ventilation biomarkers across various COPD severities in both former and current smokers, aiding model validation and data interpretation. The validated CFPD model can then be applied to predict the deposition of inhaled particles of various sizes in conducting and respiratory airways with different breathing patterns, thereby improving tailored inhalational therapies. For example, subgroup-specific inhalers or inhalational waveforms could be developed to target different regions of the lung for optimal deposition efficiency and improved clinical outcomes.

This study had several limitations. Firstly, the spatial resolution of SPECT images (3.985 mm) is lower than that of CT scans (0.5–0.7 mm), which may lead to inaccuracies in quantifying certain regions on SPECT images. Additionally, subjects were exposed to radiation during SPECT imaging, although the dose was within safety limits. Recently approved by the FDA, hyperpolarized xenon-129 magnetic resonance imaging (XeMRI) offers an alternative method for assessing lung ventilation distribution without exposing patients to ionizing radiation (Doganay et al., 2019; Peiffer et al., 2023; Kim et al., 2019; Bayat et al., 2023). Secondly, the strong correlations observed (|r| > 0.70) suggest large effect sizes, indicating potentially meaningful relationships between qCT and SPECT variables, despite the small sample size. Nonetheless, increasing the sample size and distinguishing current smokers from non-smokers is essential for achieving more robust and reliable results. Lastly, the subjects in this study effectively managed their disease over the 2-year period, with no significant changes in lung function. To gain a clearer understanding of COPD progression, it may be necessary to extend the interval between visits beyond the 2-year period.

Although this study was limited by a small sample size, our analysis suggests that SPECT ventilation imaging can effectively capture ventilation heterogeneity in COPD patients and provide complementary insights when paired with CT-based biomarkers. Specifically, SPECT images identified three distinct categories of subjects: severe COPD (Subjects 2 and 5), moderate COPD (Subjects 1, 3, and 4), and those with mild symptoms or at risk of developing COPD (Subjects 6, 7, and 8). Our findings indicate that small airway disease plays a crucial role in the heterogeneous ventilation observed in COPD patients. Additionally, the formation of hot spots in COPD could be influenced by changes in the segmental airways of the left upper lobe, alternations in airflow distribution between the upper and lower lobes, and the extent of small airway disease and emphysema. Further research into small airway disease could enhance our understanding of COPD’s diverse characteristics, aid in the identification of novel phenotypes across various imaging modalities and offer deeper insights into the disease’s progression.

## Data Availability

The original contributions presented in the study are included in the article/Supplementary Material, further inquiries can be directed to the corresponding author.
