# Supplementary material for: Longitudinal study of COPD phenotypes using integrated SPECT and qCT imaging
Source: Front Physiol. 2025 Apr 25;16:1555230. doi: 10.3389/fphys.2025.1555230 (PMC12061679; doi:10.3389/fphys.2025.1555230)
Supplement: Supplementary file 1 [file DataSheet1.pdf]

SUPPLEMENTARY MATERIAL

Content 1: Description of Pulmonary Function Tests (PFTs)

Subject went to the Pulmonary Function Lab to do several standard PFTs. These included Diffusion Capacity of the Lung for Carbon Monoxide (DLCO) and plethysmography with a Slow Vital Capacity (SVC) maneuver, and spirometry (FEV1, FVC and the FEV1/FVC ratio). The subject was given 2 puffs of albuterol and then the PFT tests were repeated. This took approximately 60 minutes. **Figure S1** shows an example of the PFT report of Subject 3 at V0. The CV_{Total}, TC_{Max}, $fSAD\%_{Total}$, and $Emph\%_{Total}$ for Subject 3 were 0.59, 7.32, 13.55%, and 18.14%, respectively, suggesting Subject 3 was a patient with moderate COPD.

All spirometry measurements in our study were conducted using the Vmax software (manufactured by CareFusion Corporation, San Diego, CA, USA), an FDA-approved system for pulmonary function testing. Tests were performed by trained, certified pulmonary function technicians with extensive clinical research experience, with the same technicians overseeing all data collection to minimize inter-technician variability. Participants were seated in the cabin of a Vmax Autobox (Body Plethysmography System, V62J) manufactured by CareFusion while performing the tests. The equipment automatically provided pulmonary function test (PFT) parameters and was consistently used across all visits for every subject. Daily calibration was performed according to manufacturer guidelines and American Thoracic Society/European Respiratory Society (ATS/ERS) standards (1), with periodic quality audits conducted throughout the study.

Spirometry

		Ref	Pre Meas	Pre % Ref	Post Meas	Post % Ref	Post % Chg
FVC	Liters	5.48	4.97	91	5.17	94	4
FEV1	Liters	4.19	(2.49)	(59)	(2.65)	(63)	6
FEV1/FVC	%	77	(50)		(51)		
FEF25-75%	L/sec	3.53	(0.79)	(22)	(0.95)	(27)	21
PEF	L/sec	10.30	(7.76)	(75)	(7.62)	(74)	-2
FET100%	Sec		16.92		14.63		-14
FIVC	Liters		3.76		3.86		3
FIF50%	L/sec		1.72		1.75		2
FVL ECode			000010		000010		
MVV	L/min						

Lung Volumes

		Ref	Pre Meas	Pre % Ref
TLC	Liters	7.50	7.21	96
VC	Liters	5.48	4.97	91
RV	Liters	2.51	2.24	89
FRC PL	Liters	4.15	3.83	92
ERV	Liters		1.29	
IC	Liters		3.38	
RV/TLC	%	36	31	
Raw	cmH2O/L/sec	1.04		
Vt	Liters		1.08	
sGaw	L/s/cmH2O/L	0.231		

Turn In: **0.003000 L**

Diffusion **Hb:**

		Ref	Pre Meas	Pre % Ref
DLCO	mL/mmHg/min	34.8	(19.3)	(55)
DL Adj	mL/mmHg/min		19.3	
VA	Liters		6.23	
DLCO/VA	mL/mHg/min/L	4.22	3.10	74
DL/VA Adj	mL/mHg/min/L		3.10	
IVC	Liters		4.92	

Figure S1. Sample PFT report of Subject 3 at V0.

Content 2: Multiscale qCT Biomarkers

The multiscale qCT biomarkers included lung structural and functional variables. The structural variables, describing the regional alterations of lung structures, included bifurcation angles (θ) between the children branches of trachea and right main bronchus (RMB), airway circularity (Cr), normalized airway wall thickness (WT^*), and normalized airway hydraulic diameter (D_h^*). Decreased θ and decreased Cr were found to be associated with airflow limitation (2) and increased functional small airway disease (3), respectively. The dimensions of wall thickness and hydraulic diameter were normalized by predicted trachea wall thickness and hydraulic diameter from healthy controls to eliminate inter-subject variability due to sex, age, and height (4). WT^*

and D_h^* reveal the effects of wall thickening and luminal narrowing on airway obstructions caused by inflammation and hyper-responsiveness, respectively. The functional variables, capturing the regional alterations of lung functions, included fractional air volume change (ΔV_{air}^F), determinant of Jacobian matrix (J), anisotropic deformation index (ADI), fraction-based small airways disease (fSAD%), fraction-based emphysema (Emph%), and tissue fraction at TLC (β_{tissue}). ΔV_{air}^F was quantified by the ratio of the lobar air-volume change to the whole lung air-volume change. The ratio of the air-volume change of the upper lobes to the air-volume change of the middle and lower lobes combined ($\Delta V_{air,UML}^F$) was also calculated. Jacobian (J) is a measure of local specific volume assessing the functional capacity of lung tissue. ADI is a measure of the magnitude of anisotropic deformation (5). Emph% and fSAD% are used to quantify the emphysematous lung tissue destruction and the extent of small airway narrowing or closure, respectively (6). β_{tissue} indicates the proportion of tissue volume for detection of tissue destruction.

Content 3: Abbreviation List of qCT Variables

The region where the qCT variable was measured is indicated as a subscript of the variable, namely $\{\text{Variable}\}_{\{\text{Region}\}}$.

Regions:

LUL: Left upper lobe

LLL: Left lower lobe

RUL: Right upper lobe

RML: Right middle lobe

RLL: Right lower lobe

Total: Total lung

RMB: Right main bronchus

LMB: Left main bronchus

BronInt: Right intermediate bronchus

TriLLB: Trifurcation of left lower lobe

sLUL: Sub-grouped segmental airways of left upper lobe

sLLL: Sub-grouped segmental airways of left lower lobe

sRUL: Sub-grouped segmental airways of right upper lobe

sRML: Sub-grouped segmental airways of right middle lobe

sRLL: Sub-grouped segmental airways of right lower lobe

Structural Variables:

θ: Bifurcation angles between the children branches of trachea and RMB

Cr: Airway circularity

WT^*: Normalized airway wall thickness

D_h^*: Normalized airway hydraulic diameter

Functional Variables:

ΔV_{air}^{F}: Fractional air volume change

J: Determinant of Jacobian matrix

ADI: Anisotropic deformation index

fSAD%: Fraction-based small airways disease

Emph%: Fraction-based emphysema

β_{tissue}: Tissue fraction at TLC

$\Delta V^{F}_{air,UML}$: The ratio of the air-volume change of the upper lobes to the air-volume change of the middle and lower lobes combined

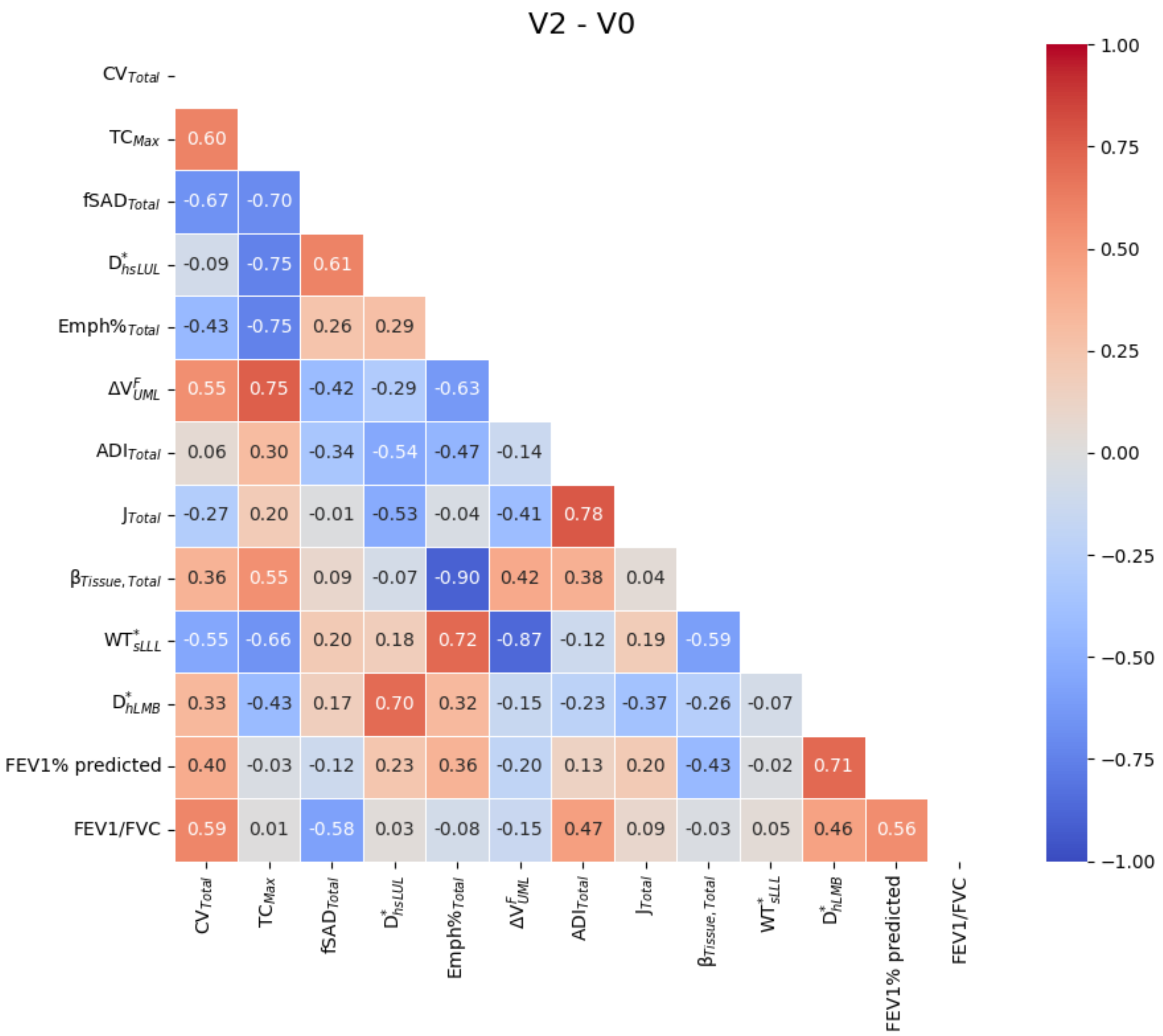

Figure S2. Correlation heatmap of 2-year change data

Table S1. Demographic data and PFT results of all the subjects at three visits. R_{aw} is the airway resistance measured by plethysmography. Ages are reported for V0.

	Gender	Age (yrs.)	BMI	Wight (kg)	Height (cm)	FEV1% predicted	FEV1/FVC	GOLD Stage	TLC (L)	FRC (L)	RV (L)	R_{aw} (cmH2O/L/Sec)
**V0**												
Subj. 1	male	80	31.6	108.0	185	73	53	2	7.48	5.03	2.98	1.18
Subj. 2	male	84	22.9	65.5	169	50	28	2	7.91	6.11	3.88	1.31
Subj. 3	male	55	25.6	88.6	186	63	51	2	7.21	3.83	2.24	1.04
Subj. 4	male	59	21.4	61.1	169	73	56	2	6.93	4.73	2.81	1.27
Subj. 5	male	59	31.6	85.0	164	36	28	3	6.79	4.79	3.24	1.75
Subj. 6	male	51	29.3	92.8	178	83	59	1	6.76	3.71	1.22	1.26
Subj. 7	female	54	27.0	75.4	167	77	71	0	5.07	2.63	1.99	1.34
Subj. 8	female	63	35.5	91.0	160	88	77	0	4.23	1.67	1.53	1.76
**Mean± SD**			28.1 ± 4.5	83.4 ± 14.4	172.3 ± 9.0	67.9 ± 16.3	52.9 ± 16.6		6.5 ± 1.2	4.1 ± 1.3	2.5 ± 0.8	1.4 ± 0.2
**V1**												
	**Gender**	**Age (yrs.)**	**BMI**	**Wight (kg)**	**Height (cm)**	**FEV1% predicted**	**FEV1/FVC**	**GOLD Stage**	**TLC (L)**	**FRC (L)**	**RV (L)**	**R_{aw} (cmH2O/L/Sec)**
Subj. 1	male		28.7	98.2	185	74	56	2	7.76	5.71	3.54	1.12
Subj. 2	male		26.0	74.3	169	39	26	3	7.76	6.02	4.4	1.4
Subj. 3	male		26.2	90.5	186	61	51	2	7.62	3.7	2.75	1.04
Subj. 4	male		20.5	58.6	169	68	58	2	7.2	4.71	3.12	1.25
Subj. 5	male		30.6	82.4	164	31	25	3	7.1	4.94	3.73	1.71
Subj. 6	male		29.3	92.8	178	78	58	2	7.37	3.71	2.06	1.26
Subj. 7	female		26.8	75.7	168	75	71	0	4.69	2.84	1.68	1.34
Subj. 8	female		33.6	86	160	97	78	0	4.23	2.21	1.34	1.69
**Mean± SD**			27.7 ± 3.6	172.4 ± 9.0	82.3 ± 11.8	65.4 ± 20.1	52.9 ± 17.8		6.7 ± 1.3	4.2 ± 1.3	2.8 ± 1.0	1.4 ± 0.2
**V2**												
	**Gender**	**Age (yrs.)**	**BMI**	**Wight (kg)**	**Height (cm)**	**FEV1% predicted**	**FEV1/FVC**	**GOLD Stage**	**TLC (L)**	**FRC (L)**	**RV (L)**	**R_{aw} (cmH2O/L/Sec)**
Subj. 1	male		28.7	98.2	185	76	53	2	7.6	5.29	3.09	1.12
Subj. 2	male		26.0	74.3	169	51	29	2	8.11	6.31	4.66	1.4
Subj. 3	male		25.4	87.8	186	62	51	2	7.6	3.82	2.68	1.06
Subj. 4	male		20.8	59.3	169	78	60	2	7.05	4.61	3.26	1.24
Subj. 5	male		30.6	82.4	164	33	28	3	7.17	5.21	3.76	1.71
Subj. 6	male		29.3	92.8	178	81	59	1	6.89	3.07	1.63	NA
Subj. 7	female		27.0	75.4	167	73	71	0	4.98	2.7	2.1	1.31
Subj. 8	female		33.6	86	160	97	78	0	4.33	1.93	1.49	NA
**Mean± SD**			27.7 ± 3.6	172.3 ± 9.0	82.0 ± 11.4	68.9 ± 18.5	53.6 ± 16.7		6.7 ± 1.3	4.1 ± 1.4	2.8 ± 1.0	1.3 ± 0.2

Table S2. The key qCT variables that contribute significantly to the factors. These variables have factor loadings with magnitudes greater than 0.6. A “(-)” indicates a negative factor loading.

F0	F1	F2	F3	F4	F5	F6
ADI_{Total}	$\beta_{tissue,\ Total}$	$D_h^*{}_{sLUL}$	$Emph\%_{Total}$	$\Delta V_{Fair, UML}$ *ΔVair, UMLF*	$WT^*{}_{sLLL}$	$D_h^*{}_{LMB}$
J_{Total}	$\beta_{tissue,\ LLL}$	$D_h^*{}_{sRLL}$	$Emph\%_{LUL}$	$\Delta V_{Fair,LUL}$ *ΔVair,LULF*	$WT^*{}_{sRUL}$	$WT^*{}_{LMB}$
J_{RLL}	$\beta_{tissue,\ RLL}$	$D_h^*{}_{sLLL}$	$Emph\%_{RUL}$	$\Delta V_{Fair,RUL}$ *ΔVair,RULF*	$WT^*{}_{sRLL}$	$D_h^*{}_{RMB}$
J_{RUL}	$\beta_{tissue,\ LUL}$	$D_h^*{}_{TriLLB}$	$Emph\%_{RLL}$	$\Delta V_{Fair,RLL}$ *ΔVair,RLLF* (-)	$WT^*{}_{TriLLB}$	
J_{LUL}	$\beta_{tissue,\ RML}$	$D_h^*{}_{sRUL}$	$Emph\%_{LLL}$	$\Delta V_{Fair,LLL}$ *ΔVair,LLLF* (-)	$WT^*{}_{sLUL}$	
J_{LLL}	$\beta_{tissue,\ RUL}$	$D_h^*{}_{sRML}$	$Emph\%_{RML}$		$WT^*{}_{sRML}$	
ADI_{LUL}	$fSAD\%_{RML}$ (-)					
J_{RML}	$fSAD\%_{Total}$ (-)					
ADI_{RUL}	$fSAD\%_{LUL}$ (-)					
ADI_{RLL}	$fSAD\%_{RUL}$ (-)					
ADI_{LLL}	$fSAD\%_{RLL}$ (-)					
ADI_{RML}	$fSAD\%_{LLL}$ (-)					

Table S3. TC% and ΔV_{air}^F of each lobe at each visit.

ID	TC%	Lobe	Visit	ΔV_{air}^F
Subj.3	0.192	LLL	V0	0.154
Subj.4	0.281	LLL	V0	0.296
Subj.5	0.183	LLL	V0	0.149
Subj.6	0.229	LLL	V0	0.153
Subj.7	0.284	LLL	V0	0.215
Subj.8	0.295	LLL	V0	0.227
Subj.3	0.272	LUL	V0	0.291
Subj.4	0.215	LUL	V0	0.200
Subj.5	0.316	LUL	V0	0.336
Subj.6	0.258	LUL	V0	0.273
Subj.7	0.183	LUL	V0	0.217
Subj.8	0.186	LUL	V0	0.221
Subj.3	0.180	RLL	V0	0.165
Subj.4	0.283	RLL	V0	0.268
Subj.5	0.191	RLL	V0	0.172
Subj.6	0.237	RLL	V0	0.035
Subj.7	0.291	RLL	V0	0.223
Subj.8	0.290	RLL	V0	0.237
Subj.3	0.037	RML	V0	0.035
Subj.4	0.059	RML	V0	0.066
Subj.5	0.075	RML	V0	0.107
Subj.6	0.055	RML	V0	0.427
Subj.7	0.049	RML	V0	0.064
Subj.8	0.062	RML	V0	0.084
Subj.3	0.319	RUL	V0	0.355
Subj.4	0.163	RUL	V0	0.170
Subj.5	0.234	RUL	V0	0.236
Subj.6	0.222	RUL	V0	0.112
Subj.7	0.193	RUL	V0	0.281
Subj.8	0.167	RUL	V0	0.230
Subj.1	0.242	LUL	V1	0.178
Subj.2	0.287	LUL	V1	0.305
Subj.3	0.311	LUL	V1	0.283
Subj.4	0.193	LUL	V1	0.212
Subj.5	0.330	LUL	V1	0.311
Subj.6	0.259	LUL	V1	0.258
Subj.7	0.183	LUL	V1	0.186
Subj.8	0.250	LUL	V1	0.180
Subj.1	0.151	LLL	V1	0.281
Subj.2	0.170	LLL	V1	0.119
Subj.3	0.126	LLL	V1	0.178
Subj.4	0.345	LLL	V1	0.268
Subj.5	0.144	LLL	V1	0.176

Subj.6	0.185	LLL	V1	0.223
Subj.7	0.244	LLL	V1	0.287
Subj.8	0.182	LLL	V1	0.297
Subj.1	0.203	RUL	V1	0.139
Subj.2	0.306	RUL	V1	0.322
Subj.3	0.417	RUL	V1	0.331
Subj.4	0.169	RUL	V1	0.173
Subj.5	0.211	RUL	V1	0.254
Subj.6	0.258	RUL	V1	0.200
Subj.7	0.237	RUL	V1	0.187
Subj.8	0.258	RUL	V1	0.170
Subj.1	0.138	RML	V1	0.109
Subj.2	0.110	RML	V1	0.121
Subj.3	0.026	RML	V1	0.038
Subj.4	0.053	RML	V1	0.064
Subj.5	0.103	RML	V1	0.073
Subj.6	0.084	RML	V1	0.095
Subj.7	0.054	RML	V1	0.049
Subj.8	0.086	RML	V1	0.063
Subj.1	0.266	RLL	V1	0.294
Subj.2	0.128	RLL	V1	0.133
Subj.3	0.121	RLL	V1	0.171
Subj.4	0.240	RLL	V1	0.285
Subj.5	0.212	RLL	V1	0.186
Subj.6	0.215	RLL	V1	0.224
Subj.7	0.282	RLL	V1	0.292
Subj.8	0.225	RLL	V1	0.291
Subj.1	0.271	LUL	V2	0.192
Subj.2	0.273	LUL	V2	0.337
Subj.3	0.365	LUL	V2	0.312
Subj.4	0.209	LUL	V2	0.235
Subj.5	0.343	LUL	V2	0.300
Subj.6	0.286	LUL	V2	0.259
Subj.7	0.205	LUL	V2	0.202
Subj.8	0.246	LUL	V2	0.195
Subj.1	0.127	LLL	V2	0.267
Subj.2	0.193	LLL	V2	0.095
Subj.3	0.113	LLL	V2	0.109
Subj.4	0.372	LLL	V2	0.245
Subj.5	0.153	LLL	V2	0.196
Subj.6	0.130	LLL	V2	0.221
Subj.7	0.231	LLL	V2	0.260
Subj.8	0.194	LLL	V2	0.283
Subj.1	0.224	RUL	V2	0.157
Subj.2	0.279	RUL	V2	0.319
Subj.3	0.401	RUL	V2	0.411

Subj.4	0.151	RUL	V2	0.179
Subj.5	0.220	RUL	V2	0.246
Subj.6	0.293	RUL	V2	0.241
Subj.7	0.267	RUL	V2	0.210
Subj.8	0.235	RUL	V2	0.163
Subj.1	0.161	RML	V2	0.112
Subj.2	0.113	RML	V2	0.124
Subj.3	0.020	RML	V2	0.034
Subj.4	0.047	RML	V2	0.063
Subj.5	0.090	RML	V2	0.066
Subj.6	0.121	RML	V2	0.069
Subj.7	0.058	RML	V2	0.057
Subj.8	0.093	RML	V2	0.055
Subj.1	0.217	RLL	V2	0.271
Subj.2	0.141	RLL	V2	0.124
Subj.3	0.100	RLL	V2	0.134
Subj.4	0.220	RLL	V2	0.279
Subj.5	0.194	RLL	V2	0.192
Subj.6	0.169	RLL	V2	0.211
Subj.7	0.238	RLL	V2	0.271
Subj.8	0.233	RLL	V2	0.304

Table S4. Additional key qCT variables.

Subject	V0	V1	V2	V1-V0	V2-V1	V2-V0
			**ADI_{Total}**			
1	0.58	0.46	0.52	-0.12	0.06	-0.06
2	0.47	0.40	0.41	-0.07	0.02	-0.06
3	0.69	0.71	0.68	0.02	-0.03	-0.01
4	0.28	0.28	0.38	0.00	0.10	0.10
5	0.52	0.51	0.43	-0.01	-0.08	-0.09
6	0.66	0.72	0.71	0.06	-0.01	0.05
7	0.37	0.36	0.46	-0.01	0.10	0.09
8	0.52	0.52	0.55	0.00	0.03	0.03
**Mean±SD**	0.51±0.13	0.5±0.15	0.52±0.11	-0.02±0.05	0.02±0.06	0.01±0.07
			**J_{Total}**			
1	1.92	1.78	1.98	-0.14	0.20	0.06
2	1.61	1.51	1.47	-0.10	-0.03	-0.14
3	2.57	2.94	2.48	0.36	-0.45	-0.09
4	1.55	1.53	1.71	-0.01	0.18	0.16
5	1.89	1.98	1.67	0.10	-0.31	-0.21
6	2.55	2.88	2.99	0.33	0.11	0.44
7	1.85	1.85	2.13	0.00	0.28	0.27
8	2.40	2.58	2.70	0.18	0.12	0.31
**Mean±SD**	2.04±0.38	2.13±0.55	2.14±0.5	0.09±0.18	0.01±0.24	0.1±0.22
			**$\beta_{tissue,\ Total}$**			
1	12.51	9.67	9.48	-2.84	-0.19	-3.03
2	9.57	6.54	7.01	-3.03	0.47	-2.56
3	7.49	7.77	8.82	0.28	1.04	1.33
4	10.71	11.18	10.43	0.47	-0.76	-0.28
5	5.40	5.88	5.40	0.47	-0.47	0.00
6	14.12	13.84	13.84	-0.28	0.00	-0.28
7	15.07	15.45	15.07	0.38	-0.38	0.00
8	11.56	10.71	10.24	-0.85	-0.47	-1.33
**Mean±SD**	10.81±3.05	10.13±3.17	10.04±3.01	-0.67±1.37	-0.09±0.55	-0.77±1.35
			**WT_{sLLL}***			
1	0.62	0.61	0.63	-0.01	0.01	0.01
2	0.57	0.62	0.57	0.05	-0.06	0.00
3	0.67	0.67	0.58	0.00	-0.09	-0.09
4	0.59	0.59	0.58	-0.01	0.00	-0.01
5	0.60	0.61	0.61	0.01	0.00	0.01
6	0.60	0.61	0.61	0.01	0.00	0.01
7	0.64	0.63	0.63	-0.01	0.00	-0.01
8	0.59	0.57	0.57	-0.02	0.00	-0.02
**Mean±SD**	0.61±0.03	0.61±0.03	0.6±0.02	0±0.02	-0.02±0.03	-0.01±0.03
			**$D_{h,LMB}$***			
1	0.73	0.75	0.72	0.02	-0.02	-0.01
2	0.63	0.63	0.60	0.00	-0.03	-0.03

3	0.71	0.70	0.68	-0.01	-0.02	-0.03
4	0.70	0.67	0.70	-0.03	0.03	0.00
5	0.57	0.56	0.56	-0.01	0.00	-0.01
6	0.68	0.63	0.60	-0.06	-0.03	-0.09
7	0.68	0.66	0.63	-0.01	-0.03	-0.04
8	0.69	0.62	0.70	-0.07	0.08	0.01
**Mean±SD**	0.67±0.05	0.65±0.05	0.65±0.06	-0.02±0.03	0±0.04	-0.03±0.03

Table S5. The correlations of the SPECT variables with fSAD%$_{Total}$ and Emph%$_{Total}$ at each visit.

	V0		V1		V2	
	**CV$_{Total}$**	**TC$_{Max}$**	**CV$_{Total}$**	**TC$_{Max}$**	**CV$_{Total}$**	**TC$_{Max}$**
**fSAD%$_{Total}$**	0.90	0.86	0.93	0.90	0.94	0.86
**Emph%$_{Total}$**	0.87	0.96	0.74	0.67	0.72	0.88

Supplementary References

1. Miller MR, Hankinson J, Brusasco V, Burgos F, Casaburi R, Coates A, et al. Standardisation of spirometry. Vol. 26, European Respiratory Journal. 2005. p. 319–38.

2. Onoe R, Yamashiro T, Handa H, Azagami S, Matsuoka S, Inoue T, et al. 3D-measurement of tracheobronchial angles on inspiratory and expiratory chest CT in COPD: Respiratory changes and correlation with airflow limitation. International Journal of COPD. 2018;13:2399–407.

3. Vasilescu DM, Martinez FJ, Marchetti N, Galbán CJ, Hatt C, Meldrum CA, et al. Noninvasive imaging biomarker identifies small airway damage in severe chronic obstructive pulmonary disease. Am J Respir Crit Care Med. 2019;200(5):575–81.

4. Choi S, Hoffman EA, Wenzel SE, Castro M, Fain SB, Jarjour NN, et al. Quantitative assessment of multiscale structural and functional alterations in asthmatic populations. J Appl Physiol. 2015;118(10):1286–98.

5. Amelon R, Cao K, Ding K, Christensen GE, Reinhardt JM, Raghavan ML. Three-dimensional characterization of regional lung deformation. J Biomech. 2011;44(13):2489–95.

6. Galbán CJ, Han MK, Boes JL, Chughtai KA, Meyer CR, Johnson TD, et al. Computed tomography-based biomarker provides unique signature for diagnosis of COPD phenotypes and disease progression. Nat Med. 2012;18(11):1711–5.
